# Pulmonary metastatic gestational choriocarcinoma following an uncomplicated term pregnancy: a case report

**DOI:** 10.1186/s13256-024-04615-y

**Published:** 2024-06-30

**Authors:** Amir Masoud Jafari-Nozad, Najmeh Jahani

**Affiliations:** 1 Student Research Committee, Birjand University of Medical Sciences, Birjand, Iran; 2 Department of Gynecology, School of Medicine, Valiasr Hospital, Birjand University of Medical Sciences, Birjand, Iran

**Keywords:** Choriocarcinoma, Case report, Metastasis, Trophoblastic neoplasm

## Abstract

**Background:**

Choriocarcinoma is a highly malignant pregnancy-related trophoblastic neoplasm, characterized by early metastasis to the lungs. Therefore, patients may manifest nongynecological symptoms owing to distant metastases. The incidence of choriocarcinoma after a term pregnancy is really rare (1/160,000 pregnancies).

**Case presentation:**

We report a case of a 20-year-old Iranian woman, gravida 2 para 1 live 1 abortion 1, who was referred to our gynecology department with sudden onset dyspnea and pain in the left hemithorax the day after her labor. The index pregnancy was without any complications. After the initial workup, the elevation of β-human chorionic gonadotropin (HCG) levels (> 1,000,000) along with the identification of clinical (vaginal lesions) and radiological evidence of distant metastases (bilateral pulmonary nodes) directed us toward pulmonary metastatic choriocarcinoma diagnosis. After the oncology consult, the etoposide, methotrexate, actinomycin D, cyclophosphamide, and vincristine chemotherapy regimen was started for the patient. She responded well to the treatment and is currently continuing her chemotherapy process.

**Conclusion:**

The prognosis of choriocarcinoma is very good if the treatment is started on time. We suggest that clinicians should consider gestational trophoblastic neoplasia in their differential diagnosis of the post-natal period complications, especially after a term and nonmolar pregnancy.

## Introduction

Gestational trophoblastic neoplasia (GTN) includes a variety of rare pregnancy-related diseases that develop during or after pregnancy, from premalignant conditions to malignant tumors. These conditions usually develop after molar pregnancy and their incidence after a full-term pregnancy is rarely reported in literature [1, 2]. Choriocarcinoma (CC) is an aggressive neoplastic disease of the chorionic membranes. This trophoblastic disease is usually suspected after postpartum hemorrhage associated with increased levels of human chorionic gonadotropin (HCG) [3–5].

CC has a very high propensity to metastasize to other organs through blood circulation, especially the lungs, brain, kidney, gastrointestinal (GI) tract, and vulvovaginal region [6]. Therefore, CC is usually detected as metastasized lesions and patients may manifest nongynecological symptoms owing to distant metastases to different organs. Pulmonary metastatic CC usually presents with dyspnea, pleural effusion, and pleuritic pain [6, 7]. The incidence of CC after a term pregnancy is really rare (1/160,000 pregnant women) [3, 8]. This study aimed to report a case of pulmonary metastatic CC after an uncomplicated term pregnancy in a 20-year-old woman, who was admitted to our hospital with sudden onset dyspnea and pain in the left hemithorax the day after her labor. The prognosis of CC is very good if the chemotherapy regimen is started on time. Therefore, we suggest that clinicians should consider GTN in their differential diagnosis of the post-natal period complications, especially after a term and nonmolar pregnancy.

## Case presentation

**Fig. 1:**
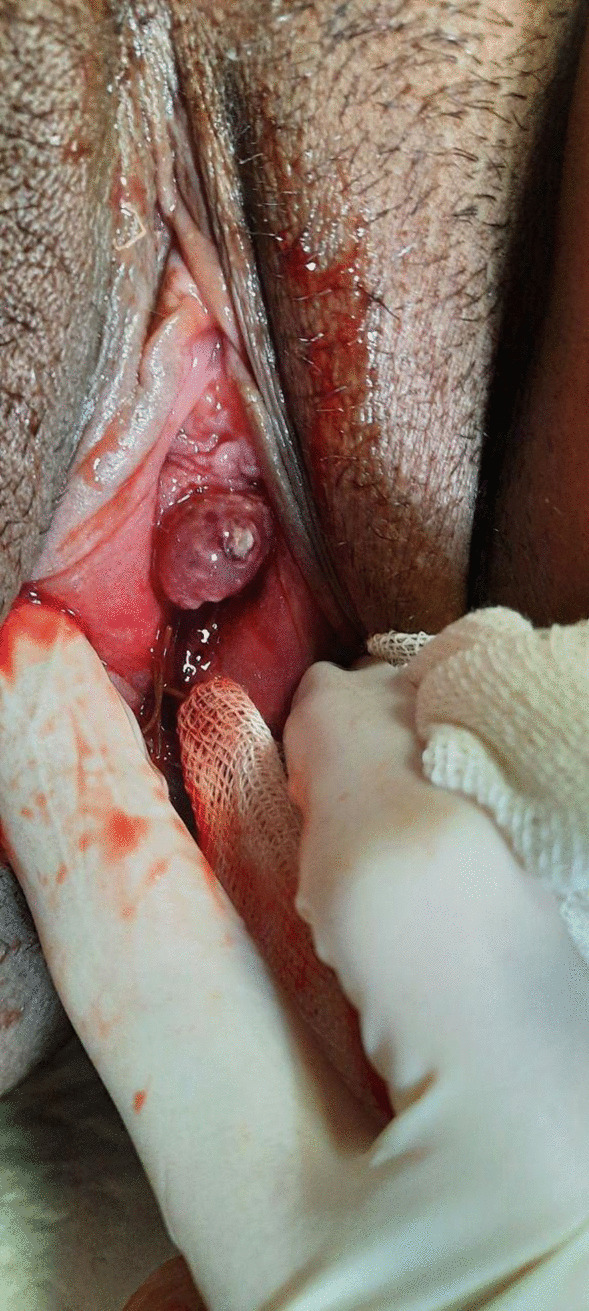
Suburethral vaginal lesion (about 1 cm) found in physical examination

The patient was a 20-year-old (gravida 2 para 1 live 1 abortion 1) Iranian female who was referred to the obstetrics and gynecology department of Valiasr Hospital, Birjand, Iran in November 2023 with sudden onset dyspnea and pain in the left hemithorax the day after her labor. The pain was severe and nonradiating. At the time of admission, the patient was agitated and was suffering from severe respiratory distress. The general examination demonstrated that the patient was tachycardiac and her left lung sound intensity was reduced (blood pressure: 136/91 mmhg, respiratory rate: 35 breaths/minute, pulse rate beats/minute: 146, body temperature: 37 ˚C, oxygen saturation: 88%). There was no vaginal bleeding but pelvic examination by a gynecologist showed a suburethral vaginal lesion (Fig. 1: 1 cm vaginal lesion), which had remained hidden owing to vaginal swelling and vulvar edema during labor.

The patient had delivered a healthy and viable infant with normal postnatal evolution yesterday (gestational age: 38 weeks + 4 days via normal vaginal delivery). The pregnancy was uneventful with no associated complications and she was discharged from the hospital the day after her labor with normal routine examinations. The patient’s obstetric history included one healthy and viable infant and one abortion at the 9th week owing to the fetus’s heart undevelopment. In the periods between her pregnancies, she had regular menses and did not mention any problems. At the time of admission, the patient’s diagnostic imaging reports during the pregnancy were reviewed and none of them had reported a placental tumor or any associated complications.

**Fig. 2:**
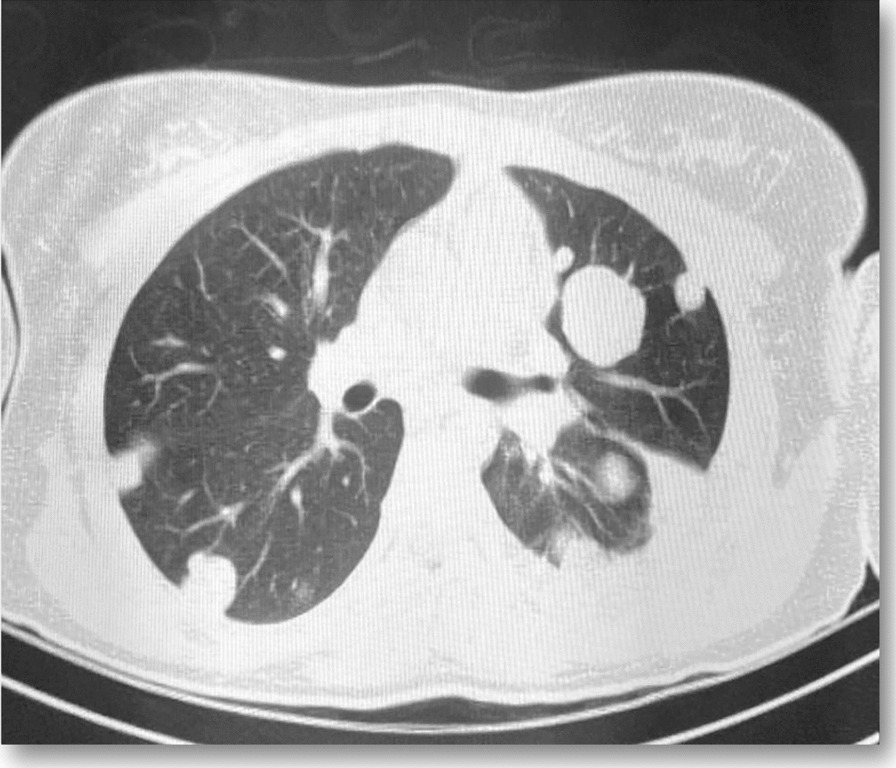
Bilateral pulmonary nodes (up to 41 mm in the right upper lobe and 34 mm in the left lung) in HRCT suggestive of metastases

**Fig. 3:**
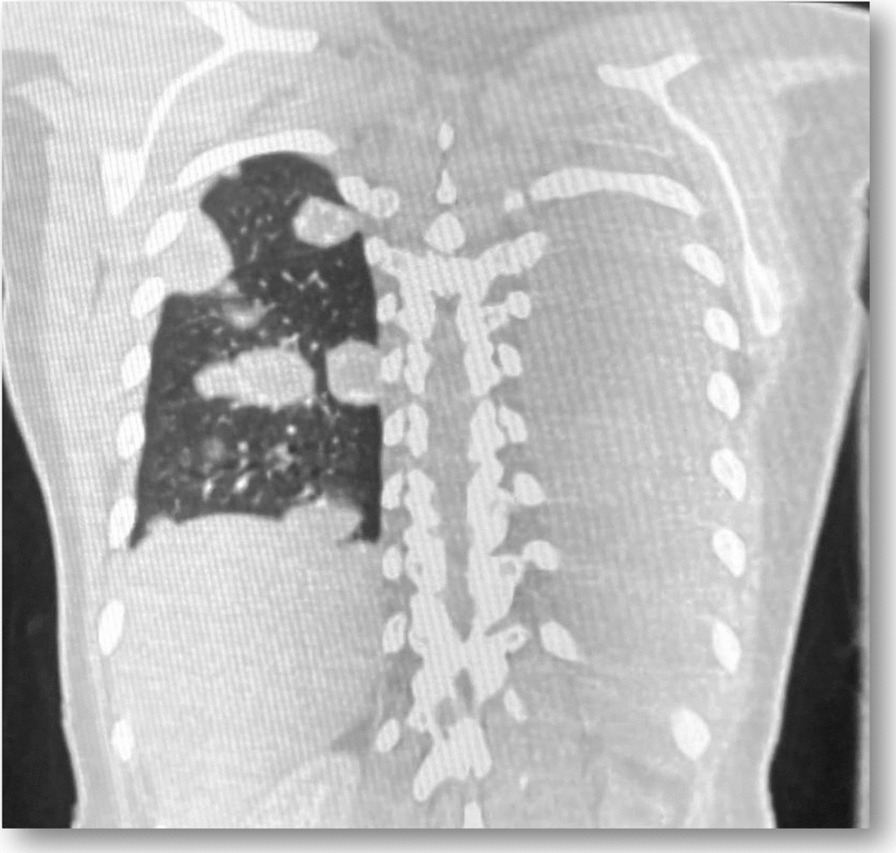
Bilateral pulmonary nodes (up to 41 mm in the right upper lobe and 34 mm in the left lung) in HRCT suggestive of metastases

Chest x-ray showed left-side hemothorax with bilateral pulmonary nodes confirmed on high-resolution computed tomography (HRCT; up to 41 mm in the right upper lobe and 34 mm in the left lung) suggestive of metastases (Figs. 2 and 3: Pulmonary nodes in HRCT). One of our main clinical suspicions was pulmonary embolism, which was rolled out by doing a computed tomographic pulmonary angiography. Blood results indicated leukocytosis [white blood cell *=(WBC) count: 14.4], anemia [hemoglobin (HB): 9.8], and a β-HCG of 1,254,800 mUI/m. An elevated β-HCG level (> 1,000,000 mUI/m) along with the identification of clinical (vaginal lesion) and radiological evidence of distant metastases (bilateral pulmonary nodes) directed us toward pulmonary metastatic CC diagnosis. The patient was admitted to the intensive care unit and closely monitored.

A transvaginal ultrasound showed a large heterogeneous mixed echoic mass in the cavity measuring 31 × 21 mm with some low-resistant peripheral vessels. To detect other potential metastatic sites, we did a chest abdomen pelvis CT, as well as a brain magnetic resonance imaging (MRI). The brain MRI showed normal intracranial appearances and no evidence of metastases. CT scan was also normal with no evidence of metastasis at spleen, kidney, liver, and GI tract.

We calculated the International Federation of Gynecology and Obstetrics (FIGO) score as below: the patient was a 20-year-old female (0 points), with a term pregnancy (2 points) 2 days ago (0 points). Her β-HCG titer before the treatment was > 1,000,000 (4 points). The physical examination showed vaginal metastasis (1 point). At the time of scoring, we were only aware of the lung metastasis (0 points), however, the imaging techniques conducted the next days showed no signs of brain, spleen, kidney, liver, and GI tract metastasis. The largest tumor size was 31 × 21 mm (1 point). In conclusion, the patient was classified as high-risk (FIGO 7 or more). After the diagnosis of metastatic CC, she was referred to and followed closely by the oncology service and a combined chemotherapy regimen [EMA/CO (etoposide, methotrexate, actinomycin D, cyclophosphamide, and vincristine)] was started for her. To reduce the risk of pulmonary hemorrhage, we considered low-dose induction chemotherapy with 100 mg/m^2^/day of intravenous etoposide and 20 mg/m^2^/day of intravenous cisplatin on days one and two every 7 days for two courses prior to starting the EMA/CO regimen. Serum HCG level monitoring demonstrated a good response to treatment. The β-HCG titer was reduced to 125 mUI/m after three cycles of chemotherapy and the patient is currently continuing her chemotherapy process.

## Discussion

GTN includes a variety of rare pregnancy-related diseases that originate from the placental tissue malignant transformation. CC is an aggressive neoplastic disease of the chorionic membranes [1]. Metastatic CC in a patient after a term pregnancy is extremely rare and the outcome is strongly dependent on clinical doubt and immediate chemotherapy regimen initiation [3, 8]. Any delay in the diagnosis can worsen the patient outcome, and we suggest that clinicians should consider CC in their differential diagnosis, especially after a term and nonmolar pregnancy. The pregnancy was uneventful in our case but CC is usually associated with pregnancy complications, including fetal hydrops, intrauterine fetal death, and fetomaternal hemorrhage.

Diagnosis is usually suspected by the patient’s history, elevated levels of β-HCG and unusual postpartum hemorrhage [4]. Katsanevakis *et al*. reported a case of a 30-year-old woman with a 5-month history of vaginal bleeding after a term and uncomplicated pregnancy, which was diagnosed as postpartum CC. The patient’s serum HCG level was 209,566 and the pelvic ultrasound showed an intrauterine mass with some vascularity (56 × 50 × 45 mm). She was planned for ten cycles of chemotherapy (etoposide, methotrexate, and actinomycin) and responded well to the treatment [3]. However, our case was admitted with dyspnea and pain in the left hemithorax. Pulmonary metastatic CC usually presents with dyspnea, pleural effusion, and pleuritic pain [7, 9]. Massive hemorrhagic pleural effusion in pulmonary metastatic CC patients is not surprising and out of mind owing to the rapid growth of the tumor and bleeding from lung metastatic lesions [10]. Therefore, the patients can become hemodynamically unstable as a result of vaginal, abdominal, or intrapulmonary massive bleeding [5]. Sapantzoglou *et al*. reported a 31-year-old female diagnosed with pulmonary metastatic CC after an uncomplicated term pregnancy (HCG > 225,000 IU/mL and an 8.5 × 7 cm heterogeneous mass in the myometrium). The patient responded well to the treatment (suction, evacuation, curettage, and six cycles of EMA/CO chemotherapy regimen) and delivered a healthy female newborn without complications after 18 months [11].

Histological examination of the placenta or endometrial curettings and biopsies can confirm the CC diagnosis. Biopsies to confirm the diagnosis is a matter of debate and controversy. Some believe that biopsy of the distant lesions is not necessary and should not be performed because of their significant vascularization and high risk of bleeding [12, 13]. Considering that the labor was uneventful and the patient became symptomatic the day after, the placenta was not kept for microscopic examination since pathological examination of the placenta is not routinely performed in uncomplicated pregnancies.

The outcome for CC, if appropriately referred to a tertiary center and immediate initiation of the chemotherapy regimen, is really good [3]. As part of FIGO staging, a prognostic score (or risk) is estimated to determine the optimal treatment course and identify the single-agent chemotherapy-resistant cases that require combination chemotherapy. The survival rate for low-risk patients (FIGO 6 or less) is nearly 100% and about 94% for people with high-risk (FIGO 7 or more) gestational CC [14–16]. The prognosis of patients is worse in cases of nonmolar pregnancy, likely because of a delay in diagnosis or advanced metastasized condition [16]. The standard treatment for GTN is chemotherapy, using a single agent or combination chemotherapy in low-risk and high-risk patients, respectively [16]. These conditions are known as a highly curable group of pregnancy‐related diseases; however, some patients will be resistant to initial chemotherapy. As a new field of investigation, the role of immunotherapy in the management of GTN needs more exploration [17]. In chemoresistant cases, the use of immune checkpoint inhibitors, including anti-programmed death-ligand 1 (PD-L1), may characterize a novel and effective therapeutic strategy. For example, pembrolizumab (anti-PD-L1) has effectively caused complete responses in about 80% of chemoresistant patients [17, 18].

## Conclusion

CC in a term pregnancy is a likely underdiagnosed and extremely rare condition. Although diagnosing can be challenging, early detection and immediate chemotherapy regimen initiation can dramatically affect the patient’s outcome. Therefore, we suggest that clinicians should consider GTN in their differential diagnosis of the postnatal period complications, especially after a term and nonmolar pregnancy.

## Data Availability

The data that support the findings of this study are available upon request from the corresponding author.
